# Orthodromic and Antidromic Snare Techniques for Left Ventricular Lead Implantation in Cardiac Resynchronization Therapy

**DOI:** 10.3390/jcm11082133

**Published:** 2022-04-11

**Authors:** Juwon Kim, Sung Ho Lee, Hye Ree Kim, Tae-Wan Chung, Ji-Hoon Choi, Ju Youn Kim, Kyoung-Min Park, Young Keun On, June Soo Kim, Seung-Jung Park

**Affiliations:** 1 Division of Cardiology, Department of Medicine, Heart Vascular Stroke Institute, Samsung Medical Center, Sungkyunkwan University School of Medicine, Seoul 06351, Korea; abcd186a@gmail.com (J.K.); hrmanse@naver.com (H.R.K.); dany3645@gmail.com (T.-W.C.); isaturni1985@gmail.com (J.-H.C.); kzzoo921@gmail.com (J.Y.K.); bkm1101@hanmail.net (K.-M.P.); yk.on@samsung.com (Y.K.O.); js58.kim@samsung.com (J.S.K.); 2 Division of Cardiology, Department of Internal Medicine, Kangbuk Samsung Hospital, Sungkyunkwan University School of Medicine, Seoul 03181, Korea; shsh96.lee@samsung.com

**Keywords:** cardiac resynchronization therapy, left ventricular lead, snare, responder

## Abstract

The snare technique can be used to overcome unsuitable cardiac venous anatomies for left ventricular (LV) lead implantation in cardiac resynchronization therapy (CRT) procedures. However, limited data exist regarding performance of the snare technique. We classified 262 patients undergoing CRT procedure into the snare (*n* = 20) or conventional group (*n* = 242) according to the LV lead implantation method. We compared the safety, efficacy, and composite outcome (all-cause death and heart failure readmission) at 3 years post-implant between the snare and conventional groups. In the snare group, all LV leads were implanted safely using orthodromic (*n* = 15) or antidromic (*n* = 5) techniques, and no immediate complications occurred including vessel perforation, tamponade, and lead dislodgement. During follow-up, LV lead threshold and impedance remained stable without requiring lead revision in the snare group. There were no significant between-group differences regarding LV ejection fraction increase (12 ± 13% vs. 12 ± 13%, *p* = 0.929) and LV end-systolic volume reduction (18 ± 48% vs. 28 ± 31%, *p* = 0.501). Both groups exhibited comparable CRT-response rates (62.5% vs. 60.6%, *p* = 1.000). The risk of primary outcome was not significantly different between the two groups (25.9% vs. 30.9%, *p* = 0.817). In patients who failed conventional LV lead implantation for CRT, the snare technique could be a safe and effective solution to overcome difficult coronary venous anatomy.

## 1. Introduction

Cardiac resynchronization therapy (CRT) has demonstrated prognostic benefits in patients with systolic dysfunction and ventricular dyssynchrony [1,2,3], and it has been incorporated into current guidelines [4,5]. However, about one-third of patients do not show improvement in symptoms or cardiac function after CRT due to patient, device, or procedure-related factors [6,7,8]. Among these factors, proper positioning of the left ventricular (LV) pacing lead at the latest activation areas of the LV is one of the major procedure-related determinants for better CRT response [9,10,11,12]. Nevertheless, LV pacing lead implantation remains technically challenging since the success rate of targeted LV lead implantation is significantly affected by the highly variable anatomy of the coronary venous system [13]. Various maneuvers and techniques have been introduced to overcome this issue [14,15].

Among the various techniques, Worley et al. first reported the LV lead implantation technique using a goose neck snare in patient with stenosis at the target vein ostium [16]. They advanced the guidewire retrograde into the target vein at the LV lateral wall via the collateral/connecting vessel and back into the coronary sinus (CS) ostium. Then, the wire was snared at the CS ostium using a goose neck snare inserted through the same subclavian sheath for insertion of the guidewire. The snared guidewire was pulled through and out of the sheath. Using a veno-venous loop, the LV lead was advanced successfully into the stenotic lateral target vein over the distal end of the guidewire while traction was maintained on the proximal end of the guidewire. Since then, there have been several case reports using the snare technique [17,18,19]. However, limited data are available regarding the performance of the snare technique compared with that of the conventional LV lead implantation method [20]. Thus, we sought to compare the safety and efficacy of the snare technique with those of the conventional method for LV lead implantation in terms of CRT procedure and outcomes.

## 2. Materials and Methods

### 2.1. Study Population

**Figure 1:**
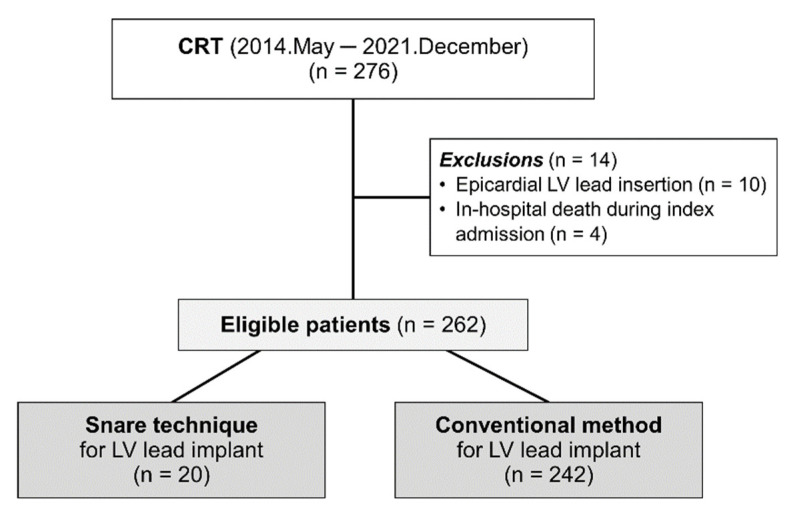
Study Flow. According to LV lead implantation method, patients were classified into the snare technique group and the conventional method group. CRT, cardiac resynchronization therapy; LV, left ventricle.

We enrolled 276 consecutive patients who underwent CRT (de novo or upgrade) for advanced heart failure (HF) at our institution between May 2014 and December 2021. All enrolled patients had LV ejection fraction (LVEF) ≤35% on transthoracic echocardiography, QRS duration ≥120 msec on 12-lead electrocardiogram (ECG), and New York Heart Association functional class II, III, or ambulatory IV despite guideline-directed optimal medical treatment for at least 3 months. We excluded patients who had undergone epicardial LV lead insertion (*n* = 10) or died during index admission (*n* = 4). Finally, a total of 262 patients were selected for the current study. According to the method of LV lead implantation, patients were classified into the snare technique group (*n* = 20) and the conventional method group (*n* = 242) (Figure 1). The institutional review board of our institution approved this study and waived the requirement for written informed consent.

### 2.2. CRT Implantation and Snare Technique

CRT devices were implanted under local anesthesia using a transvenous approach [21]. For conventional LV lead implantation, CS cannulation catheters and sub-selection catheters were used. After CS cannulation, contrast venography was performed, and the 0. 014″ guidewire was advanced to the target vein. Then, the LV lead was delivered to the target vein over the 0.014″ guidewire. When central vein obstruction was encountered, vessel patency for further procedures was secured by venoplasty using non-compliant balloons, serial dilation with different sized dilators, or extraction of previous leads. The LV leads were placed preferably into the anterolateral, lateral, or posterolateral LV walls in the left anterior oblique view and into the basal or mid-LV segments in the right anterior oblique view. However, the use of other sites was allowed at the discretion of the physician according to individual clinical or anatomical situations. Atrioventricular and ventriculo-ventricular delays were determined to show the greatest stroke volume or narrowest QRS duration during the index hospitalization before discharge.

**Figure 2:**
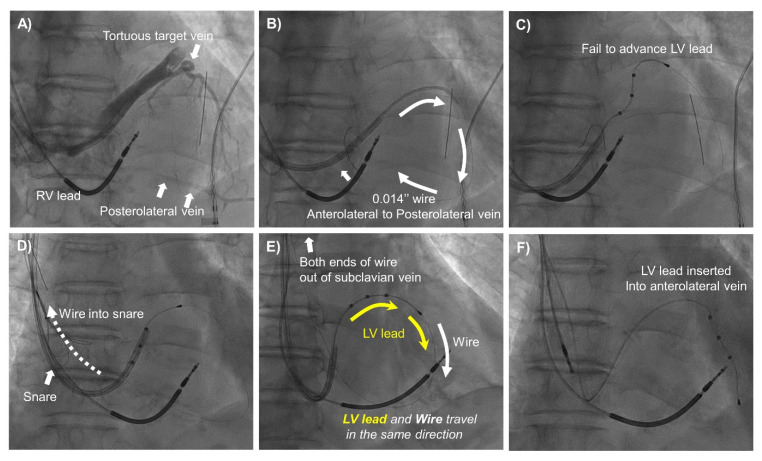
The Orthodromic Snare Technique. (**A**) Venography showed that the proximal portion of the target branch (anterolateral vein) was tortuous with a connecting vein present between anterolateral and posterolateral veins. (**B**) A 0.014″ guidewire was advanced into the anterolateral vein and back into the CS ostium via the connecting vein (white arrow). (**C**) LV lead insertion into the target branch was unsuccessful due to its tortuous course. (**D**) The distal end of the guidewire was snared at the right atrium. (**E**) The distal end of the guidewire was snared out of the sheath. The LV lead was inserted via the guidewire proximal end (yellow arrow), and they traveled in the same direction. (**F**) Successful LV lead implantation into the anterolateral vein. CS, coronary sinus; LV, left ventricle; RV, right ventricle.

**Figure 3:**
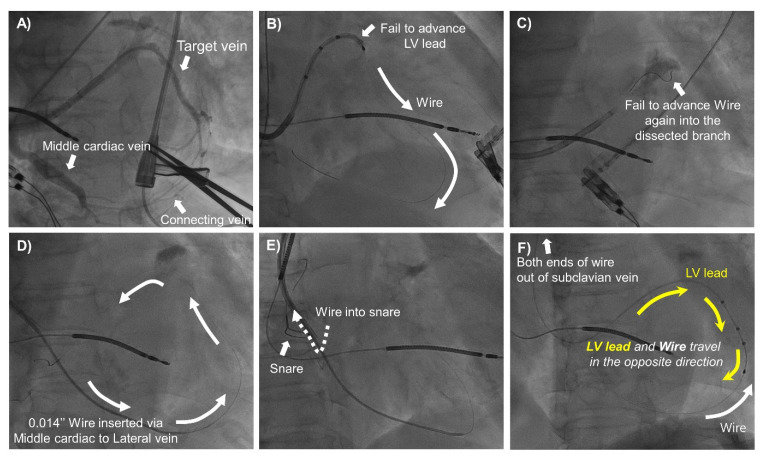
The Antidromic Snare Technique. (**A**) Venography showed the target vein (lateral) and the connecting vein to the middle cardiac vein. (**B**) Failure to advance the LV lead into the target vein. (**C**) Re-wiring into the target branch was unsuccessful due to the dissected ostium of the target vein. (**D**) The 0.014″ guidewire was advanced into the middle cardiac vein and back into the CS ostium via the connecting vein (white arrow). (**E**) The guidewire distal end was snared at the right atrium. (**F**) The distal end of the guidewire was snared out of the sheath. The LV lead was advanced into the target vein via the guidewire distal end (yellow arrow). The LV lead and guidewire traveled in opposite directions. CS, coronary sinus; LV, left ventricle.

The snare technique was tried when conventional LV lead implantation failed due to anatomical difficulties including great tortuosity, stenosis, small diameter, or ostial dissection of target veins. Additional venography was performed in a super-selected side branch using 4 or 5 Fr vein selectors (Glidecath, Terumo, Tokyo, Japan) to evaluate the presence of collateral/connecting vessels between the target and nearby veins. Two kinds of snare technique, orthodromic and antidromic, were performed, similar to the methods described by Worley et al. [15]. The ‘orthodromic’ and ‘antidromic’ snare techniques were defined as the LV lead implant skills where a 0.014″ guidewire and LV lead were inserted in the ‘same’ and ‘opposite’ directions, respectively, during the procedure (Graphic abstract, Figure 2 and Figure 3). The term of ‘antegrade insertion’ was used when the LV lead or guidewire was inserted through the ostium of the target vein toward its distal portion (Supplementary Figure S1). The term of ‘retrograde insertion’ was used when the LV lead or guidewire was inserted from the distal portion of the target vein toward its ostium (Supplementary Figure S1). Retrograde insertion is possible only when connecting veins are large enough for the LV lead to pass through.

More specifically, in the orthodromic snare technique, the 0.014″ guidewire was advanced antegrade into the target vein and then back into the CS ostium via collateral/connecting vessels. Then, the guidewire was snared around the CS ostium using an Amplatz Goose Neck snare (Medtronic, Minneapolis, MN, USA) or EN Snare (Merit Medical, South Jordan, UT, USA). The snares were usually introduced via the sheaths prepared for insertion of the right atrial or right ventricular leads. The snared distal end of the guidewire was pulled out of the sheath. Using a veno-venous loop, the LV lead was inserted at the proximal end of the guidewire and then advanced into the target vein along the guidewire, while the distal end of the guidewire was under the control of the operator (Graphic abstract and Figure 2). Conversely, in the antidromic snare technique, a 0.014″ guidewire was advanced retrograde into the target vein via the collateral/connecting vessel and then back into the CS ostium. The distal end of the guidewire was snared around the CS ostium and pulled out. Then, the LV lead was inserted at the distal end of the guidewire to the targeted vein while the tension of the proximal end of the guidewire was maintained by the operator (graphic abstract and Figure 3).

### 2.3. Data Collection and Follow-Up

Baseline characteristics, 12-lead ECG, echocardiography, CRT device analysis, and clinical outcome data were collected prospectively from our CRT registry by trained research coordinators using a standardized case report form and protocol. Patients were followed up at 3, 6, 9, and 12 months after the index procedure and biannually thereafter. Further information was collected by telephone contact or medical records, if necessary. ECG and CRT device analyses were performed at every visit. In pacing-dependent patients with previous pacemakers upgraded to CRT, paced-QRS complexes were used for assessment of baseline QRS duration and morphology. Echocardiography was performed at 6 and 12 months after the index procedure and annually thereafter. LVEF and LV end-systolic volume (LVESV) were assessed by the biplane Simpson’s method. Medical treatments for advanced HF were performed based on current guidelines [22].

### 2.4. Study Outcomes and Definitions

The primary composite outcome included all-cause death and HF readmission. Secondary outcomes were cardiac death, left ventricular assist device implantation, heart transplantation, LV lead dislodgement or failure, and individual components of the primary outcome. All deaths were considered to be cardiac deaths unless a definite noncardiac cause could be established. All end points in this study were censored at 3 years after the index procedure. The mean follow-up duration of the study population was 22.6 ± 19.9 months. The definitions of CRT responders were as follows: (1) ‘responder’ for patients with LVEF improvement from baseline ≥10% or LVESV reduction from baseline ≥15% and (2) ‘super-responder’ for patients with LVEF improvement from baseline ≥20% or LVESV reduction from baseline ≥30%.

### 2.5. Statistical Analysis

Continuous variables were analyzed using unpaired *t*-test or the Mann–Whitney rank-sum test and presented as mean and standard deviation according to distribution, which was assessed by the Kolmogorov–Smirnov test and visual inspection of Q-Q plots. All discrete or categorical variables were presented as number and relative frequency (percentage) and compared using the chi-squared test or Fisher’s exact test. In comparing follow-up echocardiography and ECG parameters between the snare and conventional groups, we analyzed the most recent results within 3 years of the index procedure. LV lead pacing threshold and impedance were compared at baseline and 3, 6, 12, and 24 months between the two groups.

The cumulative incidence of primary outcome was presented as Kaplan–Meier estimate and compared using a log-rank test or Breslow test. Multivariable Cox proportional hazard regression was used to calculate hazard ratio (HR) and 95% confidence interval (CI) to compare the risk of clinical events between the snare group and the conventional group. Multivariable Cox proportional hazard models were constructed using variables that were potentially clinically relevant: age, baseline LVEF, and baseline LVESV.

All analyses were two-tailed, and statistical significance was defined as *p* < 0.05. Statistical analyses were performed using SPSS 25.0 for Windows (SPSS-PC, Chicago, IL, USA), and R version 3.6.0 (R Foundation for Statistical Computing, Vienna, Austria).

## 3. Results

### 3.1. Baseline Characteristics

Among a total of 262 patients, the snare technique was used in 20 and the remaining 242 underwent CRT implantation without snare. Demographics, cardiovascular risk factors, and initial ECG findings including atrial fibrillation, left bundle branch block, and QRS duration were not significantly different between the snare and conventional groups (Table 1). The conventional group had a numerically higher prevalence of ischemic cardiomyopathy. The snare group showed larger baseline LVEF and smaller baseline LVESV than the conventional group. The baseline and procedural details of the snare group are presented in Supplementary Table S1. All patients in the snare group had left bundle branch block morphology and underwent CRT-defibrillator device implantation.

### 3.2. Acute Procedural Outcomes in the Snare Group

In the snare group, the snare technique was used as a rescue approach for failed LV implantation for tortuous (*n* = 11), stenotic/small-sized (*n* = 7), or dissected target veins (*n* = 2). All LV leads were deployed successfully in the lateral LV walls using orthodromic (*n* = 15) or antidromic snare technique (*n* = 5). Five antidromic snare cases were performed due to failure of antegrade-wiring into the tortuous target vein (*n* = 3) or target vein dissection that occurred during the conventional approach (*n* = 2). The mean procedure time for overall rescue CRT procedure using the snare was 178 ± 70 min. The mean procedure time tended to be longer in the snare group than in the conventional group (178 ± 70 vs. 146 ± 37 min, *p* = 0.086). However, the mean procedural time of the snare group decreased with increasing experience with the snare technique (the first half of vs. last half of cases, 206 ± 64 vs. 151 ± 69 min, *p* = 0.079) (Supplementary Table S1). Eventually, the last half of snare cases showed similar procedure time compared with the conventional group. The first half of snare cases was performed between 2014 and 2018, and the second half of cases were performed between 2019 and 2021. The mean LV pacing threshold and impedance were 1.40 ± 0.66 V and 669 ± 225 ohm, respectively. No immediate complications including vessel perforation, pericardial effusion or tamponade, pneumothorax, or pocket hematoma occurred in the snare group. Furthermore, there were no events of acute LV lead dislodgement, lead malfunction, or lead revision in the snare group.

### 3.3. Changes in LV Lead Pacing Threshold and Impedance

**Figure 4:**
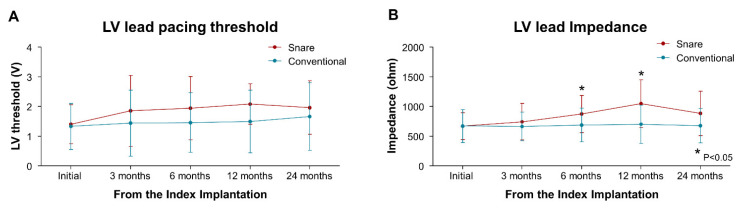
LV Lead Pacing Threshold and Impedance between the Snare and Conventional Groups. (**A**) LV lead pacing threshold and (**B**) LV lead impedance at baseline and 3, 6, 12, and 24 months after CRT implantation was compared between the snare and conventional groups. CRT, cardiac resynchronization therapy; LV, left ventricle.

During long-term follow-up, the LV lead pacing threshold of the snare group slightly increased at 3 months but then remained stable and well-maintained around 2.0 V at 0.4 ms. The LV lead pacing thresholds were not significantly different between the snare and the conventional groups at baseline or 3, 6, 12, and 24 months after the procedure (Figure 4). The LV lead impedance at 6 and 12 months were significantly higher in the snare group than in the conventional group (Figure 4). However, the LV lead impedance was well maintained within the mean range of 600 to 1050 Ω during follow-up in both groups.

### 3.4. Electrocardiographic and Echocardiographic Responses

**Figure 5:**
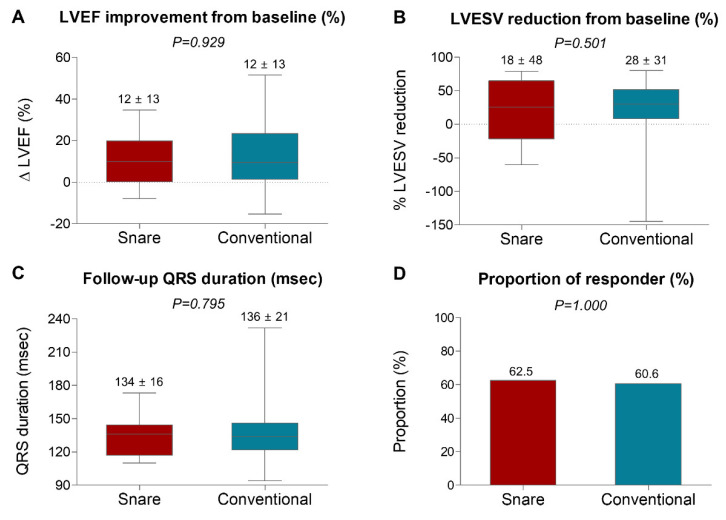
Comparison of Electrocardiographic and Echocardiographic Responses between the Snare and Conventional Groups. (**A**) Absolute increase in LVEF, (**B**) relative reduction in LVESV, (**C**) follow-up QRS duration (msec), and (**D**) responder rates (%) are compared between the snare and conventional groups. Values are mean ± standard deviation or proportion (%). In box-and-whisker plots, horizontal line indicates median value, box indicates the interquartile range, and whiskers indicate the minimum and maximum values excluding outliers. LVEF, left ventricular ejection fraction; LVESV, left ventricular end-systolic volume.

Follow-up QRS duration and QRS narrowing from baseline were not significantly different between the two groups (Table 2 and Figure 5). There were no significant between-group differences in follow-up LVEF, LVESV, and their relative changes from baseline (Table 2 and Figure 5). Furthermore, responder and super-responder rates defined by echocardiographic parameters were comparable between the snare and conventional groups (62.5% vs. 60.6%, *p* = 1.000 and 37.5% vs. 42.3%, *p* = 0.915, respectively). The detailed follow-up electrocardiographic and echocardiographic parameters of the snare group are presented in Supplementary Table S2.

### 3.5. Clinical Outcomes

**Figure 6:**
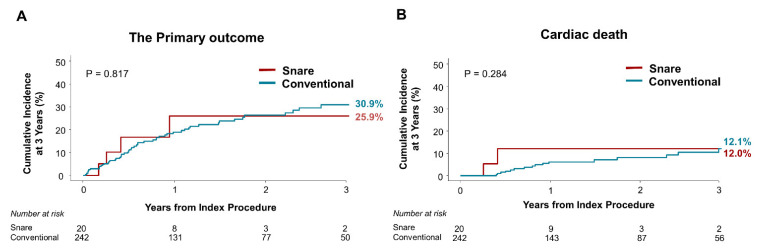
Comparison of Clinical Outcomes between the Snare and Conventional Groups. (**A**) The primary outcome (a composite of all-cause death or heart failure readmission) and (**B**) cardiac death compared between the snare and conventional groups.

The risk of primary composite outcome at 3 years was not significantly different between the snare and the conventional groups (25.9% vs. 30.9%, adjusted HR 0.831, 95% CI 0.296–2.334, *p* = 0.817) (Table 3 and Figure 6). Furthermore, both groups showed no significant differences in cumulative incidence of all-cause or cardiac death and HF re-admission. LV lead revision was required for dislodgement in 9 of 242 (5.8%) patients in the conventional group. However, there were no cases of LV dislodgement requiring lead revision or LV lead malfunction in the snare group.

## 4. Discussion

In the present study, we evaluated the feasibility of the snare technique compared with the conventional method for LV lead implantation in CRT procedure. The major findings were as follows. First, no immediate complications including vessel perforation, pericardial effusion, cardiac tamponade, or lead dislodgement or malfunction occurred in the snare group. Second, in the snare group, the LV lead pacing threshold and impedance remained stable and well-maintained without requiring lead revision during long-term follow-up. Third, the snare group showed comparable electrocardiographic and echocardiographic responses to those of the conventional group. Fourth, there was no significant difference in the risk of primary composite outcome between the snare and conventional groups.

CRT has become a standard treatment in patients with ventricular systolic dysfunction and dyssynchrony by restoring atrioventricular, inter- and intra-ventricular synchrony [8]. Positioning the LV lead in the optimal site is crucial to obtain the maximal benefit from CRT [10,11,12]. Previous studies have demonstrated that the LV lead position at the site of latest activation was associated with better clinical outcomes compared with the position at earlier activation sites [9,23]. However, LV lead placement at or near the optimal pacing sites is one of the most challenging steps of the CRT procedure because variation in the anatomy of the coronary venous system predominantly affects the final LV lead position [13,24]. Despite recent advances in CRT delivery systems, the failure rate of LV lead deployment remains high (5–10%) for many reasons, including unsuitable venous anatomy, poor lead stability, or phrenic nerve stimulation [1,25].

To date, various tools and techniques have been developed to overcome difficult coronary venous anatomy [14,15]. The snare technique is one of the most useful techniques to facilitate targeted LV lead implantation at the optimal pacing sites and first was introduced and well-established by Worley et al. [16]. This technique is applicable when connecting vessels between the target and nearby veins are available. The snare technique can be divided into orthodromic and antidromic snare techniques according to the direction in which the guidewire and LV leads travel within the body. The orthodromic snare technique could be a useful method to obtain extra support for LV lead insertion when the target vein is tortuous, stenotic, or approximately the same size as the LV lead caliber (Figure 2). On the other hand, the antidromic snare technique could be a useful solution, when antegrade wiring into the target vein is unsuccessful due to vessel dissection, severe stenosis, or tortuosity (Figure 3). There might be concerns about vessel dissection or lead damage while pushing the LV lead into tortuous and tight veins with excessive force through the veno-venous loop. However, in the present study, vessel perforation, pericardial effusion, cardiac tamponade, and lead damage did not occur in the snare group. Rather, the antidromic snare technique was used safely and effectively as a bail-out strategy in cases of ostial dissection of target vessels with the conventional method (Figure 3). Furthermore, in the snare group, the LV lead pacing threshold and impedance remained stable during long-term follow-up without lead malfunction or dislodgement. However, considering a previous report by Marques et al. in which cardiac effusion was observed in 3.2% of patients who underwent LV lead implantation using the snare technique [20], close monitoring should be performed for the possibility of vessel dissection and pericardial effusion when using the snare technique. In addition, after LV lead deployment into the target vein using the snare, the following points should be considered to remove the guidewire from the LV lead safely. (1) If the snared portion of the guidewire is bent or folded, the damaged part should be trimmed or cut before pulling back the guidewire to avoid unwanted lead dislodgement. (2) When guidewire removal is attempted in the same direction in which the guidewire was inserted, the stiff portion of the proximal end of the guidewire needs to pass through the LV lead lumen and connecting vein. Potential risk of LV lead or vein damage could be avoided or minimized by cutting off the stiff proximal end-portion of the guidewire before its removal. The present study showed that while the snare and conventional groups showed comparable efficacy in terms of electromechanical CRT response and clinical outcomes, there were no cases of LV lead dislodgement, failure, or requirement of revision during long-term follow-up in the snare group, as found in a previous report [20]. Lead dislodgement requiring revision occurred only in the conventional group. The long-term stable pacing threshold with a low dislodgement rate in the snare group might be because the LV lead was implanted more tightly into a deeper location inside the target vein by the greater back-up support provided by the snare technique. Therefore, the snare technique is advisable when an appropriate target vessel could not be used. In fact, instead of using a less adequate position, every effort should be made to ensure a real CRT which absolutely rely on a proper target vessel utilization.

Our study has several limitations. First, because the number of enrolled patients, especially in the snare group, was small, the study lacked adequate power to compare outcomes between the groups. The potential bias can influence the study outcome. Therefore, our results should be interpreted carefully. Second, the snare technique is only applicable in patients with connecting vessels between the target and nearby veins. When the rescue snare technique cannot be attempted due to lack of collateral/connecting vessels, epicardial lead implantation or conduction system pacing is an alternative for failed conventional CRT procedures. Third, CRT response is primarily determined by the final LV lead position rather than the lead implantation technique. In the present study, electromechanical CRT response and clinical outcomes were not significantly different between the snare and the conventional groups. It suggests that LV leads were properly positioned at the target area by the snare technique, although the patients had difficult coronary venous anatomy. Fourth, the snare group showed larger baseline LVEF and smaller baseline LVESV than the conventional group. Although we performed adjustments to overcome the potential bias by differences in baseline echocardiographic findings, the remaining bias might have affected study outcomes.

## 5. Conclusions

In patients who failed conventional LV lead implantation for CRT, the snare technique could be a safe and effective solution to overcome difficult coronary venous anatomy. Long-term performance of the LV lead implanted using the snare technique was well maintained without dislodgement. Furthermore, the snare technique showed comparable clinical and electromechanical CRT outcomes to those of the conventional method during long-term follow-up. Therefore, to maximize long-term CRT outcomes, the snare technique should be more aggressively considered for implanting the left ventricular lead at the optimal target site in patients with difficult anatomical barriers.

## Figures and Tables

**Table 1:** Baseline Characteristics of the Study Patients.

	Total (N = 262)	Snare Group (N = 20)	Conventional Group (N = 242)	*p* Value
**Demographics**				
Age, year	67.4 ± 11.8	67.0 ± 12.0	67.4 ± 11.8	0.883
Male	168 (64.1)	10 (50.0)	158 (65.3)	0.260
Body mass index, kg/m^2^	23.5 ± 3.6	23.0 ± 3.8	23.5 ± 3.6	0.527
**Cardiovascular risk factors**				
Hypertension	145 (55.3)	9 (45.0)	136 (56.2)	0.463
Diabetes mellitus	103 (39.3)	7 (35.0)	96 (39.7)	0.863
Chronic kidney disease *	42 (16.0)	3 (15.0)	39 (16.1)	1.000
Prior myocardial infarction	37 (14.1)	2 (10.0)	35 (14.5)	0.828
**Underlying heart disease**				0.174
ICMP	66 (25.2)	2 (10.0)	64 (26.4)	
Non-ICMP	196 (74.8)	18 (90.0)	178 (73.6)	
**ECG findings**				
Atrial fibrillation	47 (17.9)	4 (20.0)	43 (17.8)	1.000
LBBB	228 (87.0)	20 (100.0)	208 (86.0)	0.147
Initial QRS duration (msec)	169 ± 24	173 ± 26	169 ± 24	0.488
**Echocardiogram findings**				
Initial LVEF (%)	28 ± 6	30 ± 4	27 ± 7	0.004
Initial LVESV (mL)	164 ± 68	135 ± 41	167 ± 70	0.004
**Procedure type**				0.960
CRT-D	255 (97.3)	20 (100.0)	235 (97.1)	
CRT-P	7 (2.7)	0 (0.0)	7 (2.9)	
**Concurrent medications**				
Beta blocker	191 (72.9)	18 (90.0)	173 (71.5)	0.126
ACEi, ARB, ARNI	234 (89.3)	19 (95.0)	215 (88.8)	0.631
MRA	194 (74.0)	16 (80.0)	178 (73.6)	0.714

Values are presented as mean ± standard deviation or number (%). * Chronic kidney disease was defined as serum creatinine ≥2.0 mg/dL. Abbreviations: ICMP, ischemic cardiomyopathy; ECG, electrocardiography; LBBB, left bundle branch block; LVEF, left ventricle ejection fraction; LVESV, left ventricle end-systolic volume; CRT-D, cardiac resynchronization therapy-defibrillator; CRT-P, cardiac resynchronization therapy-pacemaker; ACEi, angiotensin-converting enzyme inhibitor; ARB, angiotensin II receptor blocker; ARNI, angiotensin II receptor blocker-neprilysin inhibitor; MRA, mineralocorticoid receptor antagonists.

**Table 2:** Electrocardiographic and Echocardiographic Responses between the Snare and Conventional Groups.

	Total (N = 262)	Snare Group (N = 20)	Conventional Group (N = 242)	*p* Value
**Follow-up ECG**				
Time to ECG, days	398 ± 198	347 ± 308	403 ± 185	0.436
Follow-up QRS duration, msec	136 ± 20	134 ± 16	136 ± 21	0.795
∆ QRS duration from baseline, msec	−34 ± 26	−38 ± 19	−34 ± 27	0.446
**Follow-up echocardiogram**				
Time to echocardiogram, days	423 ± 229	375 ± 323	428 ± 220	0.528
LV ejection fraction, %	40 ± 14	42 ± 14	39 ± 14	0.409
LVEF improvement from baseline, %	12 ± 13	12 ± 13	12 ± 13	0.929
LVESV, mL	119 ± 66	119 ± 75	119 ± 65	0.980
LVESV reduction from baseline, %	27 ± 33	18 ± 48	28 ± 31	0.501
Responder *	116 (60.7)	10 (62.5)	106 (60.6)	1.000
Super-responder ^†^	80 (41.9)	6 (37.5)	74 (42.3)	0.915

Values are mean ± standard deviation or n (%)**.** Abbreviations: LV, left ventricle; LVEF, left ventricle ejection fraction; LVESV, left ventricle end-systolic volume; ECG, electrocardiography. * Responder was defined as patients with LVEF improvement from baseline ≥10% or LVESV reduction from baseline ≥15%. ^†^ Super-responder was defined as patients with LVEF improvement from baseline ≥20% or LVESV reduction from baseline ≥30%.

**Table 3:** Clinical Outcomes between the Snare and Conventional Groups.

	Snare Group	Conventional Group	Unadjusted HR (95% CI)	Multivariable HR * (95% CI)	*p* Value
Patient number (N = 262)	N = 20	N = 242			
Primary outcome ^†^	25.9% (4)	30.9% (53)	0.887 (0.320–2.454)	0.831 (0.296–2.334)	0.817
All-cause death	12.0% (2)	17.2% (24)	0.684 (0.161–2.903)	0.645 (0.148–2.809)	0.604
Cardiac death	12.0% (2)	12.1% (16)	0.456 (0.104–1.992)	0.453 (0.100–2.057)	0.284
Heart failure readmission	15.6% (2)	19.3% (34)	1.181 (0.283–4.923)	1.107 (0.261–4.686)	0.819
LVAD implantation	0% (0)	4.6% (6)	NA	NA	NA
Heart transplantation	0% (0)	7.0% (9)	NA	NA	NA
LV lead dislodgement or malfunction	0% (0)	5.8% (9)	NA	NA	NA

The cumulative incidence of clinical outcomes is presented as Kaplan–Meier estimates during median follow-up of 22.6 ± 19.9 months. Number of events presented in parentheses. All *p* values were log-rank or Breslow *p* value in survival analysis. * Included covariables were age, baseline LVEF, and baseline LVESV. ^†^ The primary outcome was a composite of all-cause death or heart failure readmission. Abbreviations: LVAD, left ventricle assist device; LV, left ventricle; LVEF, left ventricle ejection fraction; LVESV, left ventricle end-systolic volume.

## Data Availability

The data that support the finding of this study are available from the corresponding author upon reasonable request.

## Supplementary Materials

The following are available online at https://www.mdpi.com/article/10.3390/jcm11082133/s1. Figure S1: Antegrade or retrograde LV lead insertion. (**A**) The term of ‘antegrade insertion’ was used when the LV lead or guidewire was inserted through the ostium of the target vein toward its distal portion. (**B**) The term of ‘retrograde insertion’ was used when the LV lead or guidewire was inserted from the distal portion of the target vein toward its ostium. Table S1. Baseline and Procedural Characteristics of the Snare Group; Table S2. Follow-up Electrocardiographic and Echocardiographic Parameters of the Snare Group.
