# Supplementary material for: Orthodromic and Antidromic Snare Techniques for Left Ventricular Lead Implantation in Cardiac Resynchronization Therapy
Source: J Clin Med. 2022 Apr 11;11(8):2133. doi: 10.3390/jcm11082133 (PMC9024966; doi:10.3390/jcm11082133)
Supplement: Supplementary file 1 [file jcm-11-02133-s001.zip › jcm-1653627-supplementary.pdf]

Supplementary Materials

Contents

Supplementary Figure S1. Antegrade or retrograde LV lead insertion

Supplementary Tables

Table S1. Baseline and Procedural Characteristics of the Snare Group

Table S2. Follow-up Electrocardiographic and Echocardiographic Parameters of the Snare Group

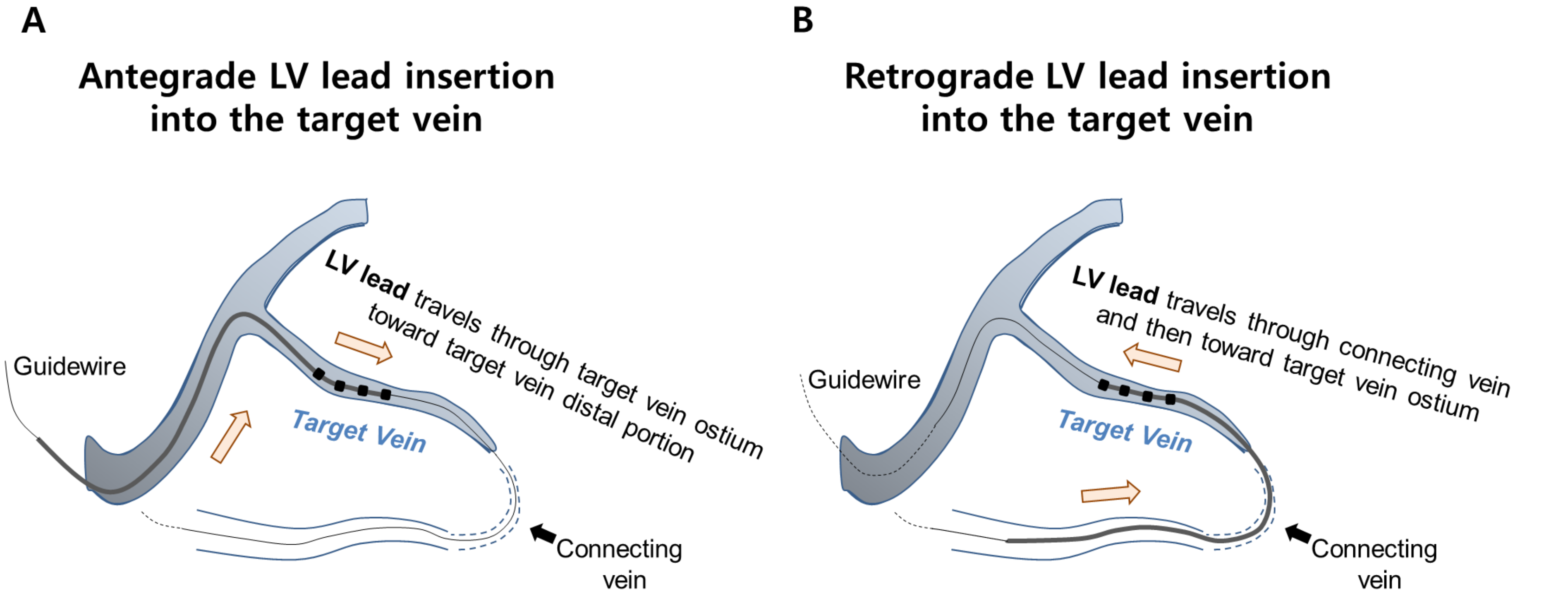

Supplementary Figure S1. Antegrade or retrograde LV lead insertion. (**A**) The term of 'antegrade insertion' was used when the LV lead or guidewire was inserted through the ostium of the target vein toward its distal portion. (**B**) The term of 'retrograde insertion' was used when the LV lead or guidewire was inserted from the distal portion of the target vein toward its ostium.

Abbreviations: LV, left ventricle

Supplementary Table S1. Baseline and Procedural Characteristics of the Snare Group.

#	**Age**(yr)**/Sex**	**Heart disease /NYHA class**	**Rhythm**	**QRSd**(ms) **/morphology**	**LV EF**(%) **/ESV**(ml)	**Procedure type**	**Target vein/ Reason for snare use**	**Snare technique**	**LV lead entry/ Final lead position ***	**Procedure time** (min)
1	58/M	DCM, II	Sinus	170, LBBB	35, 169	CRT-D (De novo)	Posterolateral / Tortuous vein	Orthodromic	Antegrade / LV mid-segment, posterolateral wall	314
2	75/M	DCM, III	Sinus	182, LBBB	32, 178	CRT-D (De novo)	Lateral / Small vein	Orthodromic	Antegrade / LV basal-segment, lateral wall	228
3	65/M	DCM, II	Sinus	157, LBBB	28, 158	CRT-D (De novo)	Lateral / Tortuous vein	Antidromic	Antegrade / LV mid-segment, lateral wall	203
4	78/F	DCM, II	AF, RV-paced	142, LBBB pattern	35, 49	CRT-D (Upgrade)	Lateral / Small, stenotic vein	Orthodromic	Antegrade / LV basal-segment, lateral wall	176
5	78/M	DCM, III	Sinus	182, LBBB	28, 186	CRT-D (De novo)	Anterolateral / Tortuous vein	Orthodromic	Antegrade / LV basal-segment, anterolateral wall	160
6	38/M	DCM, II	AF, RV-paced	246, LBBB pattern	31, 173	CRT-D (Upgrade)	Anterolateral / Tortuous vein	Orthodromic	Antegrade / LV basal-segment, anterolateral wall	214
7	72/F	DCM, III	Sinus	147, LBBB	25, 185	CRT-D (De novo)	Lateral, Vein dissection	Antidromic	Antegrade / LV mid-segment, lateral wall	231
8	74/M	ICM, III	Sinus	148, LBBB	35, 84	CRT-D (De novo)	Posterolateral, Stenotic vein	Orthodromic	Antegrade / LV basal-segment, posterolateral wall	277
9	61/M	ICM, II	AF, RV-paced	230, LBBB pattern	20, 126	CRT-D (Upgrade)	Posterolateral, Tortuous vein	Orthodromic	Antegrade / LV mid-segment, posterolateral wall	165
10	74/F	DCM, III	Sinus	158, LBBB	33, 114	CRT-D (De novo)	Anterolateral, Tortuous vein	Orthodromic	Antegrade / LV mid-segment, anterolateral wall	88
11	46/F	PICM, III	Sinus, RV-paced	170, LBBB pattern	34, 99	CRT-D (Upgrade)	Lateral, Small vein	Orthodromic	Antegrade / LV mid-segment, lateral wall	100

12	87/M	DCM, III	Sinus	166, LBBB	29, 160	CRT-D (De novo)	Anterolateral, Tortuous vein	Orthodromic	Antegrade / LV basal-segment, anterolateral wall	133
13	80/F	DCM, III	Sinus	170, LBBB	33, 138	CRT-D (De novo)	Lateral, Small vein	Orthodromic	Antegrade / LV basal-segment, lateral wall	161
14	74/F	PICM, III	Sinus, RV-paced	158, LBBB pattern	32, 120	CRT-D (Upgrade)	Lateral, Small vein dissection	Antidromic	Antegrade / LV basal-segment, lateral wall	69
15	66/M	PICM II	Sinus, RV-paced	198, LBBB pattern	28, 201	CRT-D (Upgrade)	Lateral, Small vein	Orthodromic	Antegrade / LV mid-segment, lateral wall	279
16	61/F	DCM III	Sinus	156, LBBB	29, 103	CRT-D (De novo)	Lateral / Tortuous vein	Orthodromic	Antegrade / LV basal-segment, lateral wall	235
17	55/F	DCM II	Sinus	171, LBBB	33, 104	CRT-D (De novo)	Anterolateral / Small vein	Orthodromic	Antegrade / LV mid-segment, anterolateral wall	127
18	62/F	PICM II	Sinus, RV-paced	167, LBBB pattern	29, 138	CRT-D (Upgrade)	Lateral, Tortuous vein	Antidromic	Antegrade / LV mid-segment, lateral wall	194
19	61/M	DCM III	AF	167, LBBB	27, 127	CRT-D (De novo)	Lateral, Tortuous vein	Orthodromic	Antegrade / LV mid-segment, lateral wall	137
20	70/F	PICM III	Sinus, RV-paced	174, LBBB pattern	32, 88	CRT-D (Upgrade)	Lateral, Tortuous vein	Antidromic	Antegrade / LV mid-segment, lateral wall	70

* Final LV lead position in right and left oblique views, respectively.

Abbreviations: AF, atrial fibrillation; CRT-D, cardiac resynchronization therapy-defibrillator; DCM, dilated cardiomyopathy; ICM, ischemic cardiomyopathy; LBBB, left bundle branch block; LV, left ventricle; LVEF, left ventricular ejection fraction; LVESV, left ventricular end-systolic volume; NYHA, New York Heart Association; PICM, pacing-induced cardiomyopathy; QRSd, QRS duration; RV, right ventricle

Supplementary Table S2. Follow-up Electrocardiographic and Echocardiographic Parameters of the Snare Group.

#	Time to Follow-up ECG (days)	Follow-up QRSd (ms)	Δ QRSd from baseline (ms)	Time to Follow-up echocardiogram (days)	Improvement in LVEF from baseline (%)	Relative reduction in LVESV (%)	CRT response
1	1090	142	−28	1090	−7.8	−60.4	Non-responder
2	825	142	−40	825	19.7	57.3	Super-responder
3	587	142	−15	254	8.2	28.1	Responder
4	682	114	−28	682	20	67.3	Super-responder
5	815	120	−62	815	32.2	79.2	Super-responder
6	506	146	−100	506	5.9	23.9	Responder
7	395	116	−31	192	28.2	75.0	Super-responder
8	30	130	−18	61	0		Non-responder
9	345	173	−57	346	0.7	−43.8	Non-responder
10	314	136	−22	314	34.7		Super-responder
11	279	136	−34	398	11.6	40.4	Super-responder
12	30	135	−31	78	0.1	−11.2	Non-responder
13	178	148	−22	87	−2.5	−24.8	Non-responder
14	16	125	−33	220	18.2		Responder
15	139	154	−41				
16	234	112	−44				

17	136	130	−41				
18	99	116	−51				
19	94	142	−25	64	1.6	−12.8	Non-responder
20	64	145	−29	61	17.7		Responder

Abbreviations: CRT, cardiac resynchronization therapy; ECG, electrocardiography; LVEF, left ventricular ejection fraction; LVESV, left ventricular end-systolic volume; QRSd, QRS duration.
